# Progress in Bacteriology

**Published:** 1902-07-19

**Authors:** 


### PROGRESS IN BACTERIOLOGY.

**Cystitis.**—The organism most frequently found associated with this condition is the B. coli. Albarran and Halle found it in 47 out of 50 cases, Krogius in 16 out of 20, and Melchior in 24 out of 35. As it seems possible that the organism described as B. lactis ærogenes (Escherich) is a variety of coli we may add to the above the reports of Denys who found this type in 17 out of 25 cases, and of Morelle 13 out of 17. Douglas[1] contributes a fresh series of 20 cases in 14 of which the B. coli was found. The urine was drawn off by means of a sterile glass catheter, and gelatine roll cultures immediately made. Films and cultures were also made from the centrifugalised sediment. In four cases the urine was alkaline, and in all cocci were present; in three cases secondary to prostatic enlargement, the B. coli was present in pure culture; eight cases with acid urine all showed the B. coli; one gave tubercle bacilli and one gonococci. All the foregoing cases occurred in men.

Of three cases of female patients, one was tubercular, one a mixed infection with B. coli and cocci, and the third a pure coli infection. Douglas demonstrated the fact that the introduction of micro-organisms (except the Proteus Hauser) into the bladder does not excite cystitis unless the tissues receive mechanical, chemical, or thermal injury. In the majority of cases the coli organism simply paves the way for subsequent infections. The organisms from the different cases showed marked variations in morphological and cultural characteristics, and the writer confirms the view that the B. lactis ærogenes should be classified amongst the coli groups.

**Milk Diphtheria.**—Klein[2] experimenting with sterilised milk, cream, and cheese finds that the diphtheria bacillus will grow in sterile milk at 20° C., but not at 37° C. and could not be made to grow in cream or cheese at any temperature. The streptococcus (conglomeratus) scarlatinæ, on the other hand, grows well at both 20° C. and 37° C. in milk; shows a limited growth in cream and cheese at 20° C., but none at 37° C. Dean and Todd[3] investigated a localised outbreak of diphtheria due apparently to infected milk. They found the suspected cows to be suffering from small superficial ulcers on the udders, and from these Klebs-Löffler bacilli were isolated. These organisms were virulent to guineapigs, but the guineapigs could be protected by a previous administration of diphtheria antitoxin. Healthy cows inoculated with material from the ulcers developed a papular condition of the teats, but the diphtheria bacillus was not present in these lesions.

The experiments, although not conclusive, tend to confirm the assumption that the diphtheria bacillus may excite a definite disease in cows, and that in the process of milking the milk may become infected. Cases of sore throat, closely resembling diphtheria in their clinical symptoms, may, according to Newsholme,[4] arise from milk infection. He believes that infected milk may give rise to an epidemic of scarlet fever of so mild a type that fever and sore throat are the only symptoms, even in children who have never previously suffered from the disease. The diphtheria bacillus is not found in this condition, but the streptococcus is invariably found frequently in pure culture. Milkmen's children so suffering may infect a milk supply for a long period, only a case or two appearing here and there, for the virulence is low. This is contrary to the rule of most scarlet-fever milk outbreaks in which the number of cases is large and the outbreak of brief duration. Newsholme gives an account of an outbreak of sore throats due to milk in Brighton, with a series of illustrative cases.

**Diphtheria.**—Neumann[5] records five cases of rhinitis in which virulent diphtheria bacilli were found. The symptoms, which are indistinguishable from fibrinous rhinitis, may be so mild as to escape observation, but are not infrequently found to be extremely resistant to treatment. The pseudo-diphtheria bacillus was present in some of the cases, but this association appeared in no way to modify the course of the cases. Amyot[6] examined the noses and throats of six thousand healthy persons and found the Klebs-Löffler to be present in 1 per cent. Fourteen per cent. of persons who were in more or less close contact with diphtheria cases showed diphtheria bacilli, and as no symptoms of diphtheria were to be detected the supposition is that no toxin was secreted by these organisms. The writer recommends that no person shall be regarded as free from diphtheria until negative swabbings are found on two consecutive days. To simply isolate for thirty days is unsatisfactory, this period being too short for 30 per cent. of the cases and too long for 70 per cent. The value of these findings is of course dependent upon what the writer regards as a diphtheria bacillus and in this matter there is ample scope for disagreement.

Wesbrook has classified nineteen distinct types and Hill has recently added a twentieth type of diphtheria bacillus. In the figures given by Amyot short forms are apparently included. The monograph recently published by Councilman, Mallory and Pearce[7] is a most valuable addition to our knowledge of diphtheria, being an exhaustive record of the clinical history, pathology and bacteriology of 220 fatal cases. The main point brought out by these workers is that diphtheria is a generalised bacterial infection, and not a mere local disorder. A brief abstract of their bacteriological findings will best illustrate this point. Cultures were taken from the heart's blood, liver, spleen and kidney in 153 cases of diphtheria. In the blood the Klebs-Löffler bacillus was found 7 times, in the liver 30 times, in the spleen 19 times, in the kidney 27 times. Of 56 cases of diphtheria complicated by some other disease such as scarlet fever, measles, etc., the diphtheria bacillus was found 5 times in the blood, 12 times in the liver, 7 times in the spleen, and 12 times in the kidney. In all cases only a small quantity of material, such as could be obtained on a platinum point, was examined. The writers divide the cases into those in which the bacillus was found alone and those in which there was a mixed infection, but in this abstract the totals have been added together. In one case of diphtheria complicated by scarlet fever, the diphtheria bacillus together with cocci were found in vegetations in the mitral valve. Eighty-eight cases of secondary broncho-pneumonia were examined, and the Klebs-Löffler bacillus found in 82; the same organism was also present in five out of seven cases of œdema and congestion, in three of bronchitis, and in one with slight diffuse hæmorrhage. In 22 cases of diphtheria complicated by some other disease, the broncho-pneumonic patches on examination showed the diphtheria bacillus present in 20 cases; in three out of five cases of œdema and congestion, and in one of organising pneumonia. The Klebs-Löffler bacillus was present in four out of eleven cases of abscess of the lung, and in two out of seven cases of empyema. Examination of the accessory sinuses of the nose yielded most interesting results. The maxillary antrum was thoroughly examined, and the frontal and rethmoidal cells incidentally inspected by means of Harke's section. Fifty-two cases of diphtheria are reported upon. In 21 exudation was present in both antra (five purulent; three sero-purulent; three thin, cloudy, serous fluid; one purulent fluid with membrane; and eight thin mucoid fluid), and in all but three of these the diphtheria bacillus was present on both sides. In one case the bacillus was found on one side only. Of nine cases with infection of the antrum on one side only, five were shown to be associated with the diphtheria bacillus. In one case the Klebs-Löffler bacillus was found in a diseased sphenoidal sinus. The antra were also examined in the following mixed infections:—Diphtheria with measles four cases, in three of which the diphtheria bacillus was present; diphtheria with scarlet fever seven cases, diphtheria bacillus detected in one only. Not all the cases of otitis media were examined, but the diphtheria bacillus was found in the exudate 37 times out of a total of 86 cases. In one case of thrombosis of the lateral sinus, one of the subperiosteal abscess of the parietal bone, in four of suppurating lymph-nodes, and in one of retropharyngeal abscess the diphtheria bacillus was found.

[1] Scot. Med. Jour., Feb. 15, 1902. [2] Local Gov. Brd. 29th Ann. Rep., 1901. [3] Jour. of Hyg., April 1, 1902. [4] Ibid. [5] Cent. f. Bakt., Jan. 25, 1902. [6] Canad. Tract. and Rev. Toronto, December, 1901. [7] "Diphtheria" Councilman, Mallory and Pearce.

